# Synthesis of Chain-End Functional Polydienes Using Diene Comonomer Bearing Boronic Acid Masked with Diaminonaphthalene

**DOI:** 10.3390/molecules27249007

**Published:** 2022-12-17

**Authors:** Ryo Tanaka, Yuina Kuwabara, Yuushou Nakayama, Takeshi Shiono

**Affiliations:** Department of Applied Chemistry, Graduate School of Engineering, Hiroshima University, Higashihiroshima 739-8527, Japan

**Keywords:** diene polymerization, boronic acid, chain-end functionalization

## Abstract

Diene comonomers bearing boronic acid masked with 1,8-diaminonaphthalene (dan) were applied to copolymerization with isoprene or butadiene using neodymium borohydride complex as a catalyst. The comonomers were tolerant to excess modified methylaluminoxane (MMAO) and thus were applicable to the polymerization system using MMAO. On the other hand, the corresponding pinacol borate was highly reactive toward MMAO, and no incorporation into the obtained polymer was observed. A ^13^C NMR microstructural analysis of the hydrogenated copolymer revealed that all of the comonomers were located at the chain end. Further functionalization using the boron moiety at the polymer chain end was also investigated.

## 1. Introduction

*Cis*-1,4 polyisoprene and polybutadiene are important rubber materials in industry because of their excellent physical properties [1]. These polymers are often used as a composite with other inorganic materials such as silica and carbon, and improving their affinity is, therefore, important for material use. The end-functionalization of a polymer with polar functional groups is one of the most popular ways to enhance the miscibility between polymers and inorganic additives [2].

Post-functionalization, such as olefin metathesis [3] or hydroboration [4], is the conventional way to introduce functional groups to the polymer bearing a terminal C=C double bond, although the latter is not applicable for the selective end-functionalization of polydienes. On the other hand, in situ chain-end functionalization in coordination polymerization has recently been vigorously investigated, and some are applied to diene polymerization. 

A coordinative chain transfer polymerization (CCTP) using a neodymium catalyst can be applied to functionalize the initiating chain ends of both *trans*-1,4 and *cis*-1,4 polydienes. Precise designs of chain transfer reagents, such as functionalized alkylmagnesium or alkylaluminum, successfully led to the introduction of amines and thioethers in the initiating chain end [5,6]. In the functionalization of *cis*-1,4 polydienes with organoaluminum reagents, the end-functionalization efficiency strongly depends on the structure of alkylaluminums because there are competitive chain transfers of aminoalkyl groups and non-functionalized alkyl groups [7]. 

Terminating chain ends can be functionalized by quenching the polymerization with various electrophiles. In a *cis*-1,4-specific polymerization using neodymium carboxylate catalyst, various end-capping reagents, including epoxides, ketones, and imines, are tested, and almost quantitative end-functionalization with hydroxyl or amino groups is achieved [8]. A combination of CCTP with functionalized chain transfer reagents and end-capping reagents such as silanes, epoxides, and ketones give telechelic polydiene [9]. Some vinyl monomers would be possible candidates for end-functionalization because they can be inserted into a propagating polymer chain end, which bears a metal-carbon bond. Most recently, almost quantitative functionalization was achieved with vinylsilane by using this strategy [10]. 

Recently, incorporating boronic acid moieties into polymers has received much attention, especially in view of biomedical applications, because boronic acid can bind to other polar functional groups via dynamic covalent bonds [11]. Moreover, boronic acids are bench-stable, easier to handle, and can be converted into various functional groups. A method for end-capping polydienes with boronic acid moieties would thus be of importance, but no examples are available in the current state. Some coordination–insertion copolymerization of 1,3-dienes and monomers bearing boronic acid esters using Ni(II) catalyst systems is reported [12,13]. 

In this context, we conceived to develop a new method for the end-functionalization during the coordination polymerization of 1,3-dienes using diene comonomers bearing boronic acid moieties as an end-capping reagent. Here, the design of the monomer for the successful incorporation of boronic acid and the characterization of the resulting polymer are described.

## 2. Results and Discussion

**Scheme 1:**
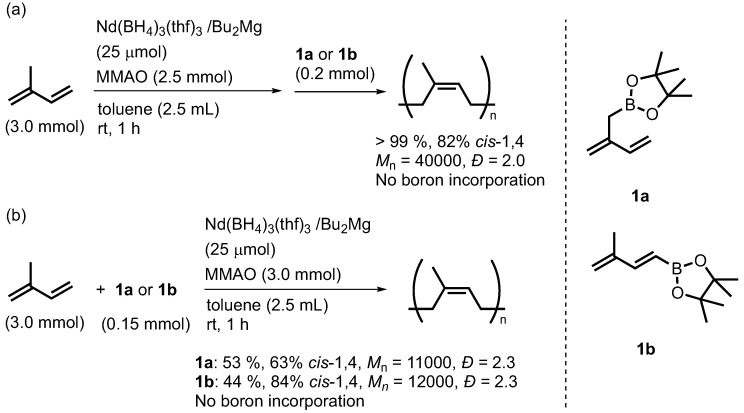
Attempt for the chain-end functionalization into polyisoprene using boronic acid ester comonomers **1a** and **1b** via (**a**) post-functionalization and (**b**) copolymerization.

The highly stereospecific polymerization of dienes, including several copolymerizations, is mainly achieved by coordination polymerization using lanthanide catalysts [14,15,16,17,18]. Therefore, the post-functionalization of polyisoprene propagated from the Nd catalyst system was investigated. We have previously shown that the Nd(BH_4_)_3_(thf)_3_/Bu_2_Mg/MMAO system successfully promotes the *cis*-1,4-specific living polymerization of isoprene and butadiene. The stereospecificity depends on the ratio of the Mg and Al reagents [19,20]. We thus first attempted the end-functionalization of the living polyisoprene prepared from this catalyst system with dienes bearing pinacol ester **1a** and **1b**. However, the incorporation of **1a** and **1b** was not observed (Scheme 1a). The copolymerization of isoprene and **1a**/**1b** was also investigated, but homopolyisoprene was obtained (Scheme 1b). No polymerization activity of **1b** toward the conventional neodymium carboxylate catalyst system is also reported [18]. 

The unsuccessful post-functionalization or copolymerization results of **1a** and **1b** may be attributed to the high reactivity of boronic acid esters with organoaluminum compounds. Alkylaluminums, including MMAO, which is fundamentally used in coordination-insertion polymerization systems, are known to cause transmetalation between boronic acid and its esters [21]. Such boron–carbon bond cleavage can be prevented by masking boronic acid with a nitrogen-containing reagent, such as 1,8-diaminonaphthalene (dan). The Lewis acidity on the boron atom is reduced by the electron donation from the nitrogen atom [22]. For the same reason, styrenic monomers containing B–N bonds in the aromatic rings undergo coordination polymerization using titanium and scandium catalysts activated by alkylaluminums [23,24]. 

**Scheme 2:**
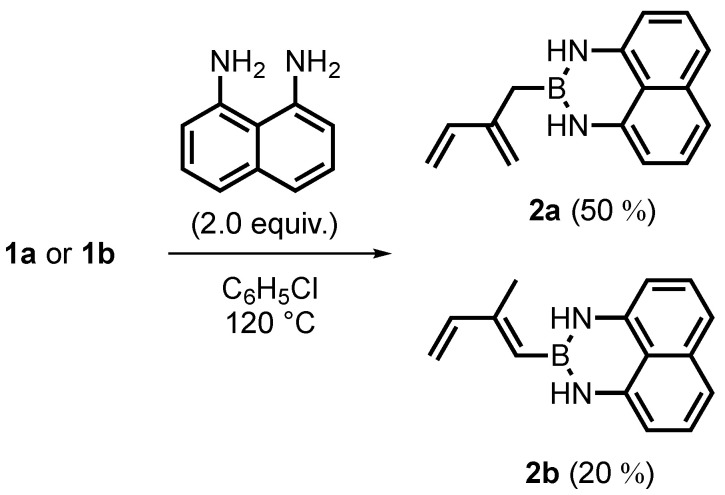
Synthesis of boron diamide comonomers **2a** and **2b**.

**Scheme 3:**
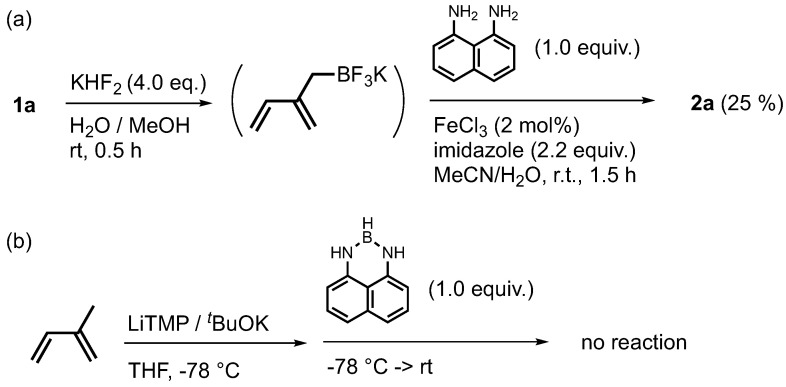
Unsuccessful attempts for the preparation of **2a**: (**a**) a synthetic route via trifluoroborate salt, (**b**) direct functionalization of potassium isoprenide.

We therefore next designed comonomers **2a** and **2b** bearing boronic acid diamide. These compounds were synthesized by the ester-amide transformation from the corresponding pinacol esters **1a** and **1b** (Scheme 2) [25]. The transamidation of **1a**/**1b** and dan proceeded slowly in chlorobenzene at 120 °C. After 24 h, the starting material was completely consumed and gave **2** in moderate yields. An attempt to synthesize **2a** via potassium trifluoroborate salt was not successful. A reaction of the salt prepared from **1a** and KHF_2_ and dan in acetonitrile/water mixture in the presence of iron(III) chloride gave **2a** in a very low yield, probably because the corresponding trifluoroborate salt and/or unprotected boronic acid is highly unstable (Scheme 3a). The direct installation of dan-masked boron to synthesize **2a** was also unsuccessful. The isoprenyl nucleophile prepared from Schlosser’s base and naphthalene-1,8-diaminatoborane was not reacted (Scheme 3b). 

**Figure 1:**
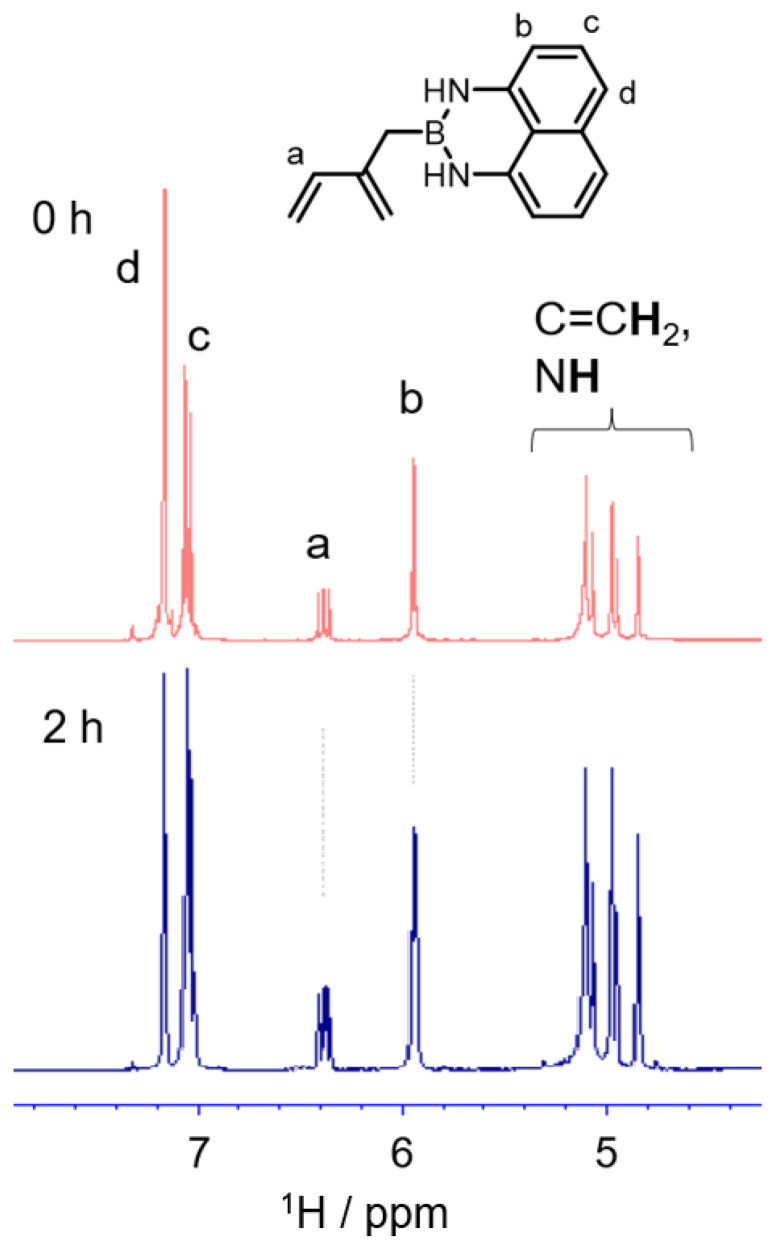
^1^H NMR spectrum of comonomer **2a** reacted with excess MMAO (500 MHz, in C_6_D_6_).

The reactivity of boron-containing comonomers toward organoaluminum compounds was confirmed by NMR studies. When pinacol ester **1a** was reacted with excess MMAO at room temperature in C_6_D_6_, 40% of **1a** was decomposed after 1 h and almost completely consumed after 24 h. In the ^11^B NMR spectrum, a new signal other than **1a** was observed at 86 ppm, assigned to trialkylborane (see Figure S8 in the Supplementary Materials). Therefore, **1a** was decomposed via transmetalation between the C-Al bond of MMAO. This result well explains our hypothesis about the unsuccessful polymerization using **1a** and **1b**. On the other hand, **2a** was intact in the presence of excess MMAO for 2 h (Figure 1). Even when the sample was heated to 45 °C for 5 h, only 22% of **2a** was decomposed and converted to trialkylborane via transmetalation (See ^11^B NMR spectrum in Figure S11). These results indicated that the dan-masking strategy of boronic acid, which was effective for the olefinic monomers, is also applicable to diene monomers.

The obtained boronic acid amide comonomer and isoprene were copolymerized using neodymium borohydride catalyst systems (Table 1). The purity of comonomer **2a** or **2b** was very sensitive to the polymerization results. Generally, the boronic acid amide is obtained as an orange solid immediately after purification but gradually turns dark brown after storage for several weeks on the bench. This is probably because the naphthalene-derived moiety was oxidized, although the ^1^H NMR spectrum did not show a significant conversion, and the comonomer at this state does not promote polymerization with satisfactory reproducibility. Therefore, comonomers **2a** and **2b** used for the polymerization should be stored in nitrogen and darkness. In addition, to minimize the effect in the catalyst preparation of the Nd/Mg/Al ternary system, a stock solution was prepared prior to the investigation. The polymerization activity of this stock solution was confirmed after storage for several weeks in a −30 °C freezer.

The copolymerization of Isoprene and **2a** gave a copolymer with moderate yield (run 1). However, the homopolymerization of **2a** did not proceed at the same condition, showing that the successive insertion of **2a** is not possible (run 2). Comonomer **2b**, of which boron atom is directly substituted on vinyl carbon, did not copolymerize at the same condition, and isoprene homopolymer was obtained, probably because **2b** is highly electron-deficient and difficult to coordinate to the neodymium center (run 3). Polymerization in the absence of MMAO, a known condition to promote the *trans*-specific polymerization of isoprene, did not proceed even at higher temperatures (run 4). The copolymerization of butadiene and **2a** also proceeded (run 5). The ^13^C NMR spectrum analyses showed that these polymerizations proceeded in a moderate *cis*-stereospecificity. The main reason for the decrease in *cis*-specificity was probably the coordination of nitrogen atoms to the metal center. The ratio of the *cis*-sequence was also decreased when butadiene polymerization was conducted in the presence of alkylboronic acid naphthalene diamide (run 6). The lower *cis*-specificity in run 6 than butadiene/**2a** copolymerization is probably because of the lower steric effect of boronic acid amide used in run 6 than **2a**. 

**Scheme 4:**
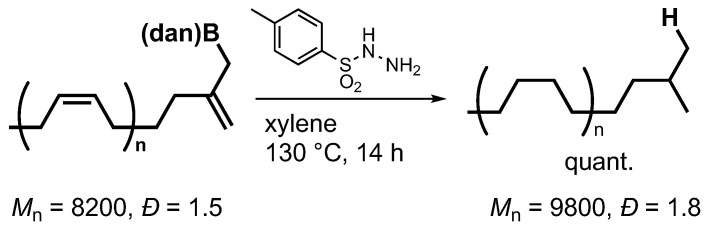
Hydrogenation of the copolymer of butadiene and **2a**.

**Figure 2:**
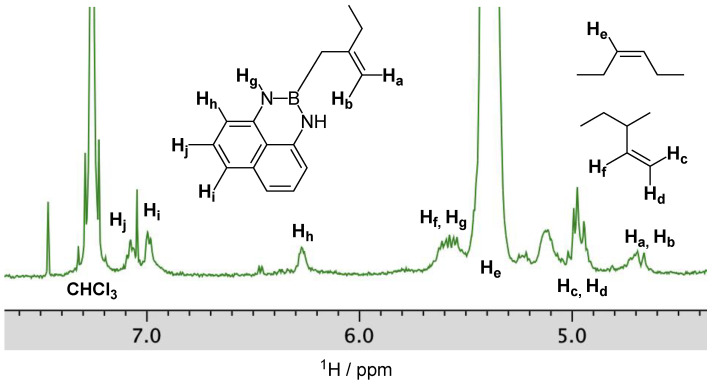
The ^1^H NMR spectrum of butadiene/**2a** copolymer obtained in Table 1, run 5 (500 MHz, in CDCl_3_).

The ^1^H NMR spectrum of the isoprene/**2a** copolymer, collected immediately after the precipitation of the reaction mixture into acidic methanol, showed the presence of three characteristic signals at 7.1, 7.0, and 6.3 ppm. These signals are assigned to the diaminonaphthalene skeleton, indicating that the comonomer was successfully incorporated into the obtained polymer. The ^11^B NMR of the copolymer showed a weak broad signal at 32 ppm, showing the presence of boronic acid amide. These signals cannot be observed after prolonged contact with acidic methanol (6 h), probably because of the deprotection of naphthalene diamide. The average polymerization degree of the polyisoprene used here calculated from *M*_n_ determined by GPC is 3100/68 = 45. By comparing the integral ratio of signals assigned to the polyisoprene main chain and **2a**, it was revealed that one **2a** molecule is inserted into one polymer chain on average (Figure S12 in supporting information). The ^1^H NMR spectrum of butadiene/**2a** showed three characteristic signals at 7.1, 7.0, and 6.3 ppm, which are observed in the isoprene/**2a** copolymer (Figure 2). Moreover, a signal with the same integral ratio appeared at 4.7 ppm, showing the formation of a 1,1-disubstituted olefinic structure. This peak indicated that **2a** was always incorporated in a 3,4-specific manner. Further structural characterization was performed in the hydrogenated copolymer. Heating the polymer solution in the presence of excess *p*-toluene sulfonyl hydrazide [26] successfully gave hydrogenated polybutadiene in an almost quantitative yield, although all the incorporated boron moiety was removed by the protodeboration side-reaction (Scheme 4). 

**Figure 3:**
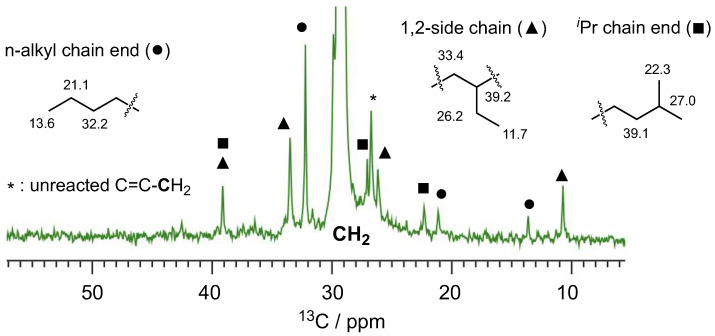
The ^13^C NMR spectrum of hydrogenated polybutadiene obtained in Scheme 4 (125 MHz, in C_2_D_2_Cl_4_).

**Figure 4:**
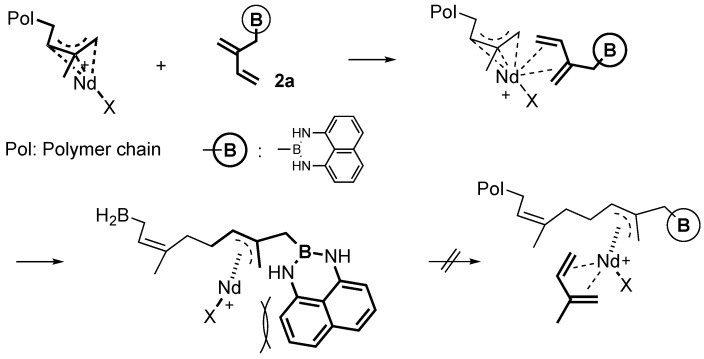
Termination of diene polymerization by **2a**.

Three characteristic signals were observed in the ^13^C NMR spectrum of the hydrogenated polybutadiene at 39.1, 27.0, and 22.3 ppm (Figure 3). These signals are assigned to terminal isopropyl groups [27], obtained by the hydrogenation and protodeboration of boronic acid diamide incorporated into the polymer chain end. On the other hand, signals assigned to internal isopropyl groups (44, 31, and 19 ppm) [28], which would be formed by the hydrogenation of internal boronic acid, were not observed. The boron functionality is therefore only incorporated into the chain end, and no further chain propagation proceeded after the insertion of comonomer **2a**. Considering that the homopolymerization of **2a** did not proceed, this is probably because the bulkiness of naphthalene diamide prevented the further coordination of the incoming monomer. Moreover, the signals of the *n*-alkyl chain end, which are derived from Bu_2_Mg, were observed at 32.2, 21.1, and 13.6 ppm with almost the same intensity with those of the isopropyl chain end. Any other chain ends were not observed, and these results indicated that the copolymerization was initiated by the alkylation of the neodymium center with Bu_2_Mg and terminated by the insertion of comonomer **2a**. Termination with **2a** occurred probably because the **2a** inserted into the polymer chain end in 3,4-manner is bulky enough to prevent the further coordination of the monomer (Figure 4).

## 3. Experimental Section

### 3.1. General

All manipulations were performed under an atmosphere of nitrogen using standard Schlenk line techniques. Modified methylaluminoxane (MMAO, 2.0 M solution in toluene) was generously donated by Tosoh-Finechem Co. (Shunan, Japan). Dry toluene and THF were purchased from Kanto Chemical Co. Inc. (Tokyo, Japan) and purified by passing through solvent purification columns. Silica gel column chromatography was performed on silica gel N60 (spherical neutral, particle size 63–210 μm, Kanto Chemical Co. Inc.). Boronic acid pinacol esters **1a** and **1b** were synthesized according to previously reported procedures [12,29]. Other materials were used as received.

Briefly, ^1^H, ^11^B, and ^13^C NMR spectra were recorded in tetrachloroethane-*d*_2_ or chloroform-*d* on a Varian system 500 NMR spectrometer. The obtained spectra were referenced to the signal of a residual trace of the partially protonated solvents [^1^H: *δ* = 7.26 ppm (CHCl_3_) and 5.91 ppm (C_2_DHCl_4_)], solvents [^13^C: *δ* = 73.8 ppm (C_2_D_2_Cl_4_), *δ* = 77.1 ppm (CDCl_3_)], or the signal of external standard [^11^B: 0 ppm (BF_3_·Et_2_O)]. The molecular weights of polymers were determined by gel permeation chromatography (GPC) on a Tosoh HLC-8320 chromatograph (*T* = 40 °C; eluent: THF) or a Tosoh HLC-8321GPC/HT chromatograph (*T* = 140 °C; eluent, *o*-dichlorobenzene) calibrated with polystyrene standards (concentration of the injected polymers: 2–3 mg/mL; injection volume: 0.2 mL). 

#### 3.1.1. Synthesis of Naphthalene Diamide Monomer **2a**

In a 20-mL Schlenk flask, boronic acid pinacol ester **1a** (1.62 g, 8.3 mmol) and 1,8-diaminonaphthalene (2.67 g, 16.6 mmol) were dissolved into chlorobenzene (8.3 mL) and stirred at 120 °C for 24 h. The solvent was removed under reduced pressure, and the residue was purified by silica gel column chromatography (hexane:toluene = 2:1, *R*_f_ = 0.68). In total, 976 mg (50%) of dark orange solid was obtained. ^1^H NMR (in CDCl_3_, 500 MHz), *δ*: 7.10 (2H, dd, ^3^*J*_H-H_ = 8 Hz, 8 Hz), 7.01 (2H, d, ^3^*J*_H-H_ = 8 Hz), 6.49 (1H, dd, ^3^*J*_H-H_ = 18 Hz, ^3^*J*_H-H_ = 10 Hz), 6.29 (2H, d, ^3^*J*_H-H_ = 8 Hz), 5.64 (2H, br), 5.24–5.02 (4H, m), 1.99 (2H, s). ^13^C NMR (in CDCl_3_, 125 MHz), *δ*: 144.0, 141.0, 139.4, 136.3, 127.5, 119.6, 117.5, 116.7, 114.6, 105.6 (ipso carbon for boron atom was not detected). ^11^B NMR (in CDCl_3_, 160 MHz), *δ*: 30.6. APCI-MS, Calcd for C_15_H_16_BN_2_: 235.14011 [M + H], Found: 235.14041.

#### 3.1.2. Synthesis of Naphthalene Diamide Monomer **2b**

In a 20-mL Schlenk flask, boronic acid pinacol ester **1b** (521 mg, 2.6 mmol) and 1,8-diaminonaphthalene (833 mg, 5.3 mmol) were dissolved into chlorobenzene (5.0 mL) and stirred at 110 °C for 15 h. The solvent was removed under reduced pressure, and the residue was recrystallized from hexane solution at −30 °C. In total, 121 mg (20%) of dark orange crystal was obtained. ^1^H NMR (in CDCl_3_, 500 MHz), *δ*: 7.13 (2H, dd, ^3^*J*_H-H_ = 8 Hz, 8 Hz), 7.04 (2H, d, ^3^*J*_H-H_ = 8 Hz), 6.90 (1H, d, ^3^*J*_H-H_ = 18 Hz), 6.34 (2H, d, ^3^*J*_H-H_ = 8 Hz), 5.78 (2H, br), 5.71 (d, 1H, ^3^*J*_H-H_ = 18 Hz), 1.94 (3H, s). ^13^C NMR (in CDCl_3_, 125 MHz), *δ*: 146.5, 143.1, 141.3, 136.5, 127.7, 119.9, 119.3, 117.6, 105.8, 18.1 (*ipso* carbon for boron atom was not detected). ^11^B NMR (in CDCl_3_, 160 MHz), *δ*: 28.0. APCI-MS, Calcd for C_15_H_16_BN_2_: 235.14011 [M + H], Found: 235.14005.

#### 3.1.3. NMR Study on the Comonomer Degradation

Approximately 5 mg of the diene monomer was dissolved into C_6_D_6_ (0.5 mL) in an NMR tube. To this solution, MMAO solution in toluene (2.0 M, 0.10 mL, 200 μmol) was added and stored at room temperature. The resulting solution was analyzed with ^1^H and ^11^B NMR spectroscopy.

#### 3.1.4. Preparation of Neodymium Catalyst Stock Solution

In a 20-mL Schlenk flask, Nd(BH_4_)_3_(thf)_3_ (81 mg, 200 μmol) was charged in the nitrogen atmosphere and suspended with a toluene solution of butadiene (2.4 M, 0.42 mL, 1.0 mmol). A solution of Bu_2_Mg (in heptane, 0.27 M, 0.74 mL, 200 μmol) was added to the suspension and stirred until the solid was completely dissolved. MMAO in toluene (2.0 M, 10 mL, 20 mmol) was added to the solution and stirred at room temperature for 20 min, turning the color of the solution from blue to yellow. The resulting catalyst solution (18 mM) can be stored under the nitrogen atmosphere at −30 °C for several weeks.

#### 3.1.5. Copolymerization of Butadiene and Boronic Acid-Containing Comonomer

As a representative procedure, the copolymerization of butadiene and **2a** (Table 1, run 2) is introduced here. In a 20-mL Schlenk flask, **2a** (117 mg, 0.50 mmol) was weighed and dissolved into a toluene solution of butadiene (2.4 M, 4.2 mmol, 10 mmol). To this solution, MMAO in toluene (2.0 M, 0.25 mL, 0.50 mmol) was added and stirred for 5 min. A stock solution of the Nd catalyst described above (18 mM, 1.4 mL, 25 µmol of Nd) was added to the stirring solution at room temperature to start the polymerization. After 2 h, the reaction mixture was poured into 50 mL of MeOH containing 1 mL of concentrated HCl to precipitate the polymer. The polymer was immediately collected by filtration, washed with water and methanol, and dried for 6 h under vacuum at room temperature. A total of 48 mg of functional polybutadiene was obtained as a brownish, rubber-like solid.

#### 3.1.6. Hydrogenation of Boron-Functionalized Polybutadiene

The polybutadiene, of which the chain end is functionalized with boronic acid amide **2a**, (*M*_n_ = 8100, *Đ* = 1.6, 25 mg) and *p*-toluene sulfonyl hydrazide (81 mg) were charged in a 20-mL Schlenk flask and dissolved in xylenes (2 mL). The solution was heated to 130 °C and stirred for 18 h. The resulting mixture was poured into methanol, and the precipitated polymer was collected by filtration. After washing with methanol and drying under a vacuum condition, 24 mg (quantitative yield) of the brownish polymer was recovered. 

## 4. Conclusions

The successful incorporation of diamide-masked boronic acid functionality at the chain end of polydienes has been achieved by the copolymerization of isoprene or butadiene with the newly synthesized comonomer, **2a**. Comonomer **2a** was much more tolerant toward the transmetalation between MMAO than the corresponding pinacol borate, **1a**. Microstructure analysis of hydrogenated copolymer by ^13^C NMR revealed that comonomer **2a** was never incorporated into the internal polymer chain but only into the chaine end, giving the chain-end functionalization polydienes in high selectivities and high yields.

## Figures and Tables

**Table 1:** Chain-end functionalization of polydienes with boronic acid diamide monomers **2a** and **2b**.

Run	R	Mg/Nd	B(dan)	Time (h)	Yield (%)	*M*_n_^a^ (10^3^)	*Đ* ^a^	Number of B per Polymer Chain ^b^	*cis*:*trans*:3,4 ^c^
1	Me	1	**2a**	1	37	3.1	2.6	1.2	64:19:17
2 ^d^	Me	1	**2a**	1	0	- ^e^	- ^e^	- ^e^	- ^e^
3	Me	2	**2b**	1	13	3.3	2.9	0	71:10:19
4 ^f^	Me	2	**2a**	2.5	0	- ^e^	- ^e^	- ^e^	- ^e^
5	H	1	**2a**	2	18	8.2	1.5	1.6	76:21:3
6 ^g^	H	1	none	1	30	6.7	1.3	- ^e^	65:33:2
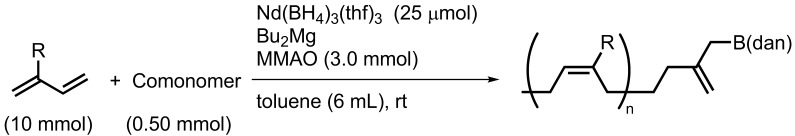									

^a^ Determined by GPC calibrated with polystyrene standards. ^b^ Determined by the boron content and molecular weight of the polymer. ^c^ Determined by NMR. ^d^ Homopolymerization of **2a**. ^e^ Not determined. ^f^ The polymerization was conducted in the absence of MMAO and at 45 °C. ^g^ 0.15 mmol of ^*i*^C_3_H_7_B(dan) was added.

## Data Availability

All data included in this study are available upon request by contact with the corresponding author.

## Supplementary Materials

The following supporting information can be downloaded at: https://www.mdpi.com/article/10.3390/molecules27249007/s1, Figure S1: ^1^H NMR spectrum of compound **2a** (500 MHz, in CDCl_3_); Figure S2: ^13^C NMR spectrum of compound **2a** (125 MHz, in CDCl_3_); Figure S3: ^11^B NMR spectrum of compound **2a** (160 MHz, in CDCl_3_); Figure S4: ^1^H NMR spectrum of compound **2b** (500 MHz, in CDCl_3_); Figure S5: ^13^C NMR spectrum of compound **2b** (125 MHz, in CDCl_3_); Figure S6: ^11^B NMR spectrum of compound **2b** (160 MHz, in CDCl_3_); Figure S7: ^1^H NMR spectrum after the reaction of **1a** and excess MMAO (500 MHz, in C_6_D_6_); Figure S8: ^11^B NMR spectrum after the reaction of **1a** and excess MMAO (160 MHz, in C_6_D_6_); Figure S9: ^1^H NMR spectrum after the reaction of **2a** and excess MMAO for 2 h at room temperature (500 MHz, in C_6_D_6_); Figure S10:^1^H NMR spectrum after the reaction of **2a** and excess MMAO for 5 h at 45 °C (500 MHz, in C_6_D_6_); Figure S11: ^11^B NMR spectrum after the reaction of **2a** and excess MMAO for 5 h at 45 °C (160 MHz, in C_6_D_6_); Figure S12: ^1^H NMR spectrum of isoprene/**2a** copolymer obtained in Table 1, run 1 (125 MHz, in CDCl_3_); Figure S13: ^13^C NMR spectrum of isoprene/**2a** copolymer obtained in Table 1, run 1 (125 MHz, in CDCl_3_); Figure S14: ^11^B NMR spectrum of isoprene/**2a** copolymer obtained in Table 1, run 1 (160 MHz, in CDCl_3_); Figure S15: ^1^H NMR spectrum of butadiene/**2a** copolymer obtained in Table 1, run 5 (500 MHz, in CDCl_3_); Figure S16: ^13^C NMR spectrum of butadiene/**2a** copolymer obtained in Table 1, run 5 (125 MHz, in CDCl_3_); Figure S17: ^11^B NMR spectrum of butadiene/**2a** copolymer obtained in Table 1, run 2 (160 MHz, in CDCl_3_); Figure S18: ^1^H NMR spectrum of hydrogenated butadiene/**2a** copolymer (500 MHz, in C_2_D_2_Cl_4_); Figure S19: Full ^13^C NMR spectrum of hydrogenated butadiene/**2a** copolymer (500 MHz, in C_2_D_2_Cl_4_).

## Sample Availability

Samples of the new compounds and polymers are available from the authors.
