# Supplementary material for: Synthesis of Chain-End Functional Polydienes Using Diene Comonomer Bearing Boronic Acid Masked with Diaminonaphthalene
Source: Molecules. 2022 Dec 17;27(24):9007. doi: 10.3390/molecules27249007 (PMC9780943; doi:10.3390/molecules27249007)
Supplement: Supplementary file 1 [file molecules-27-09007-s001.zip › molecules-2088818-supplementary.pdf]

Supporting Information

Synthesis of chain-end functional polydienes using diene comonomer bearing boronic acid masked with diaminonaphthalene

Table of Contents

NMR spectra of new compounds	2
Reactivity test of comonomers toward MMAO	4
NMR spectra of obtained copolymers	6

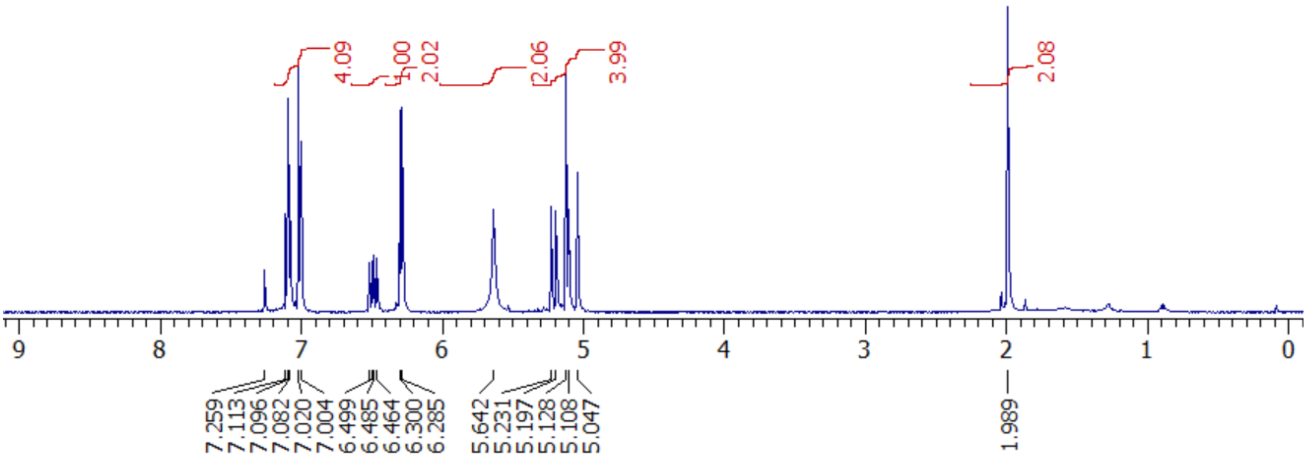

Figure S1. ^{1}H NMR spectrum of compound **2a** (500 MHz, in $CDCl_3$).

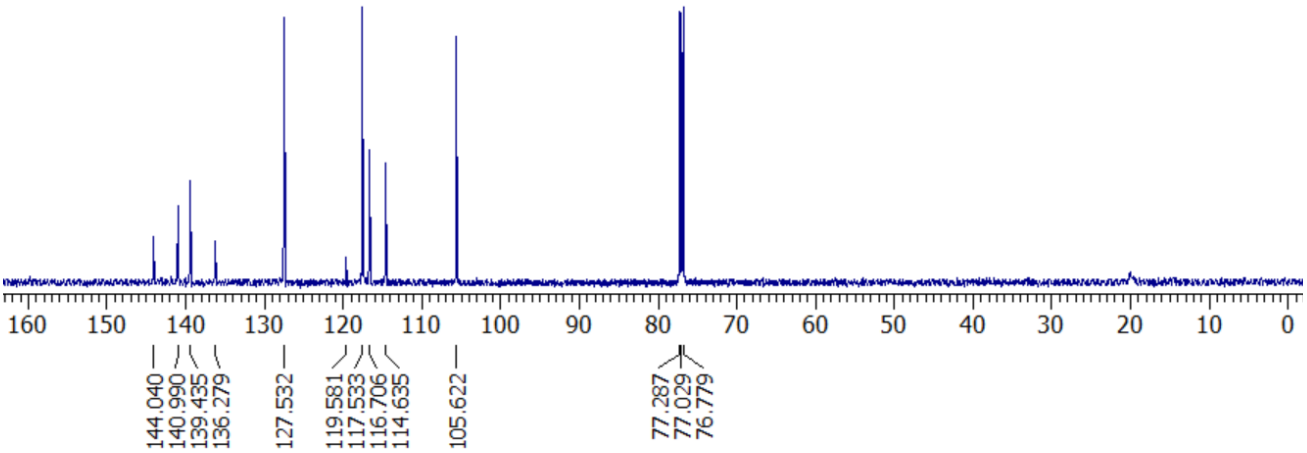

Figure S2. ^{13}C NMR spectrum of compound **2a** (125 MHz, in $CDCl_3$).

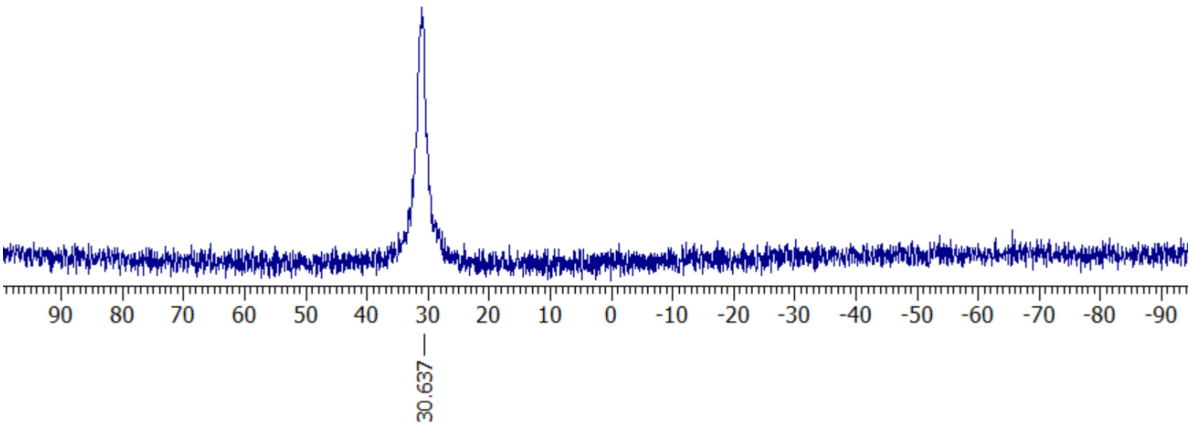

Figure S3. ^{11}B NMR spectrum of compound **2a** (160 MHz, in $CDCl_3$).

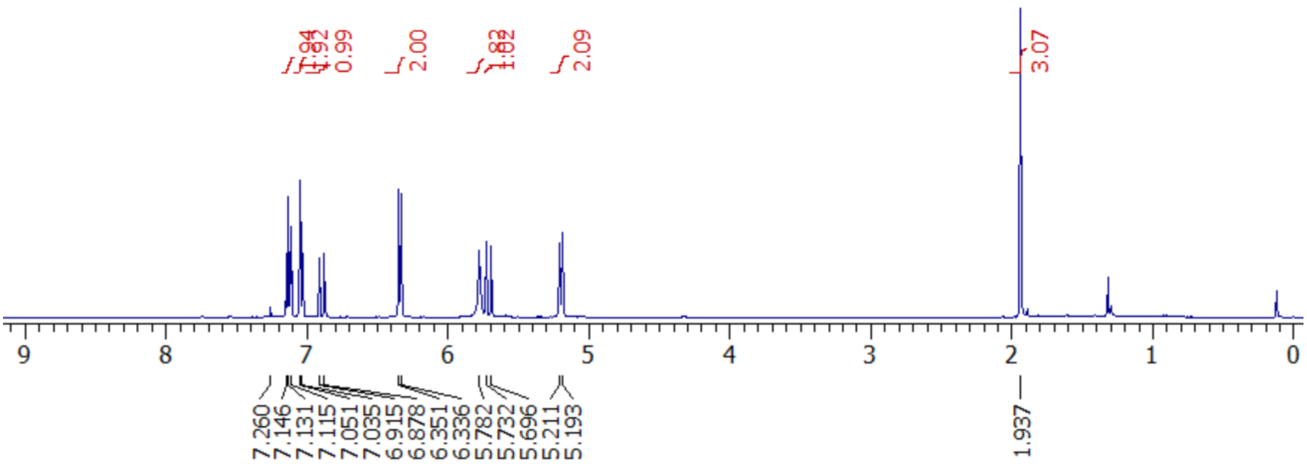

Figure S4. ^{1}H NMR spectrum of compound **2b** (500 MHz, in $CDCl_3$).

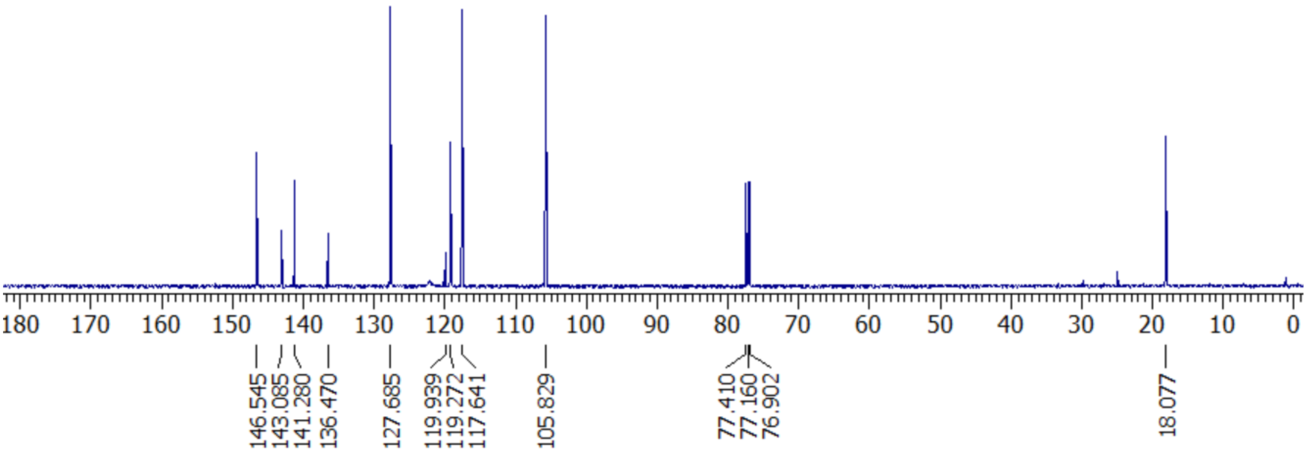

Figure S5. ^{13}C NMR spectrum of compound **2b** (125 MHz, in $CDCl_3$).

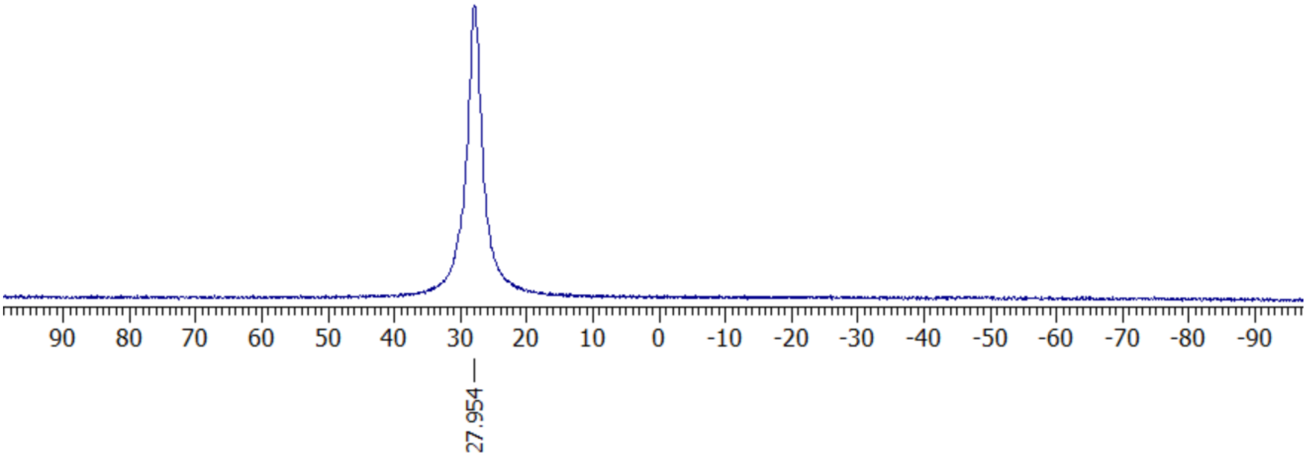

Figure S6. ^{11}B NMR spectrum of compound **2b** (160 MHz, in $CDCl_3$).

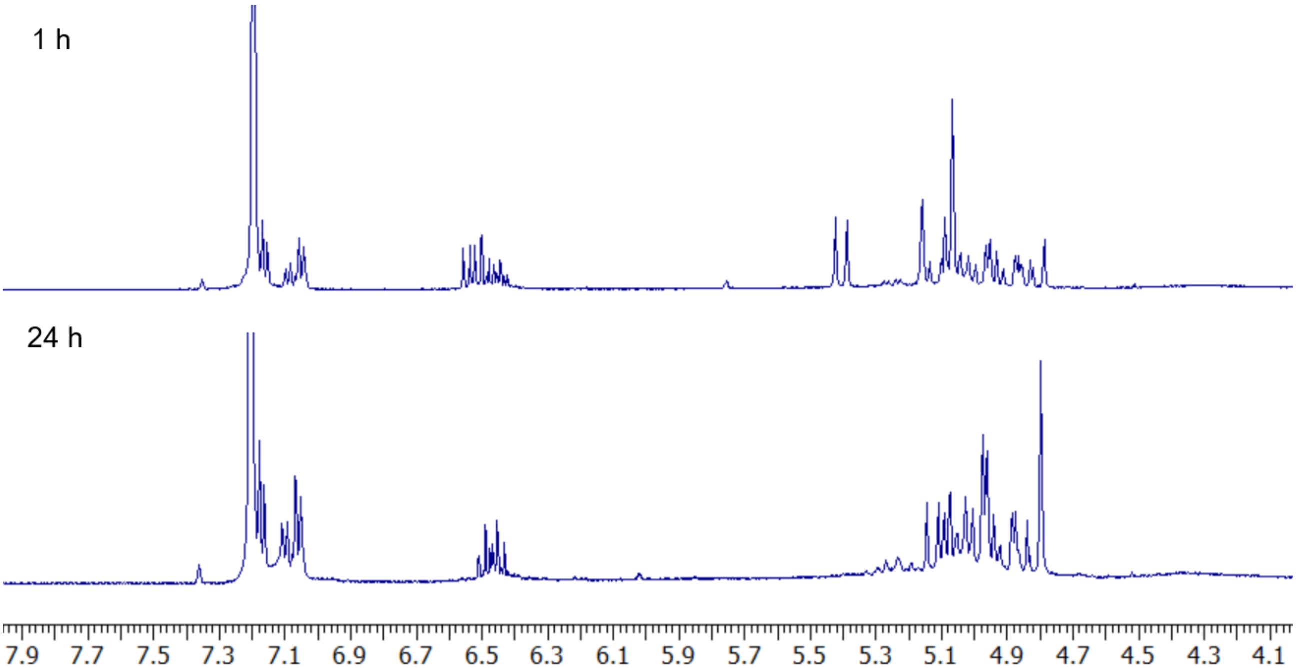

Figure S7. ^{1}H NMR spectrum after the reaction of **1a** and excess MMAO (500 MHz, in C_6D_6).

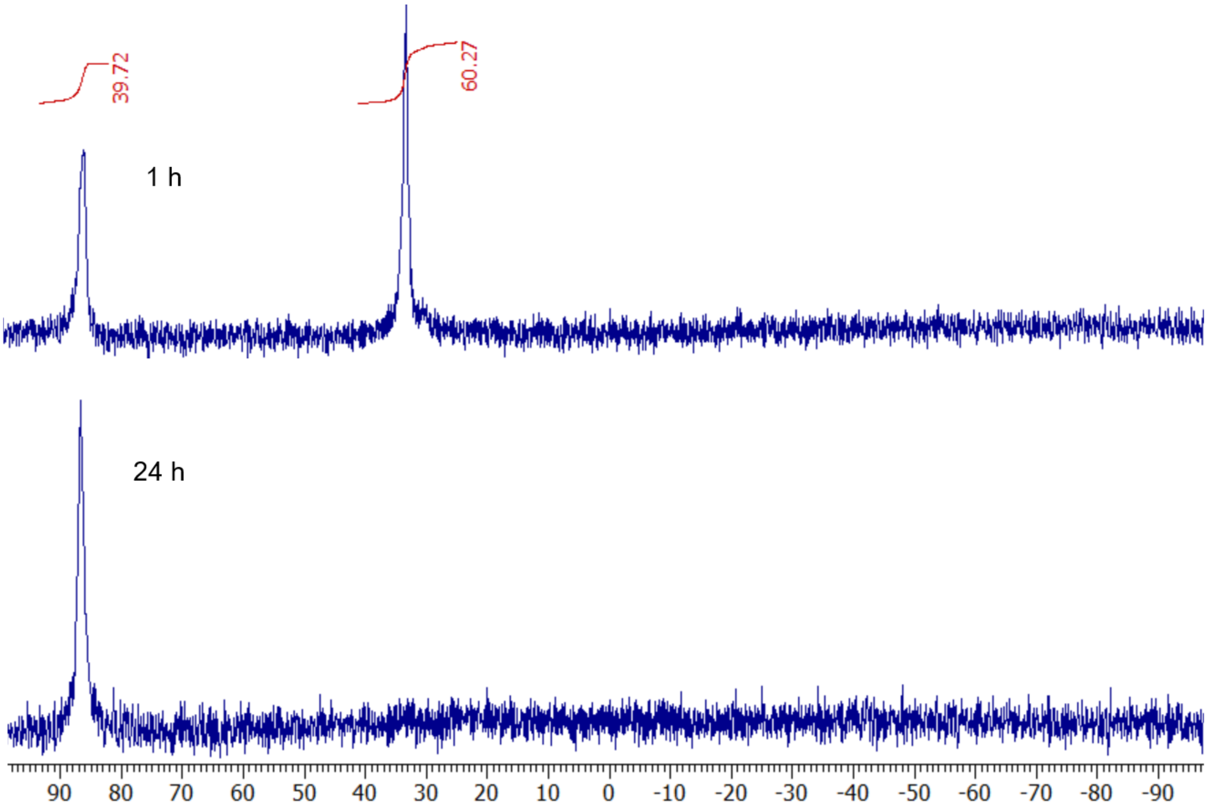

Figure S8. ^{11}B NMR spectrum after the reaction of **1a** and excess MMAO (160 MHz, in C_6D_6).

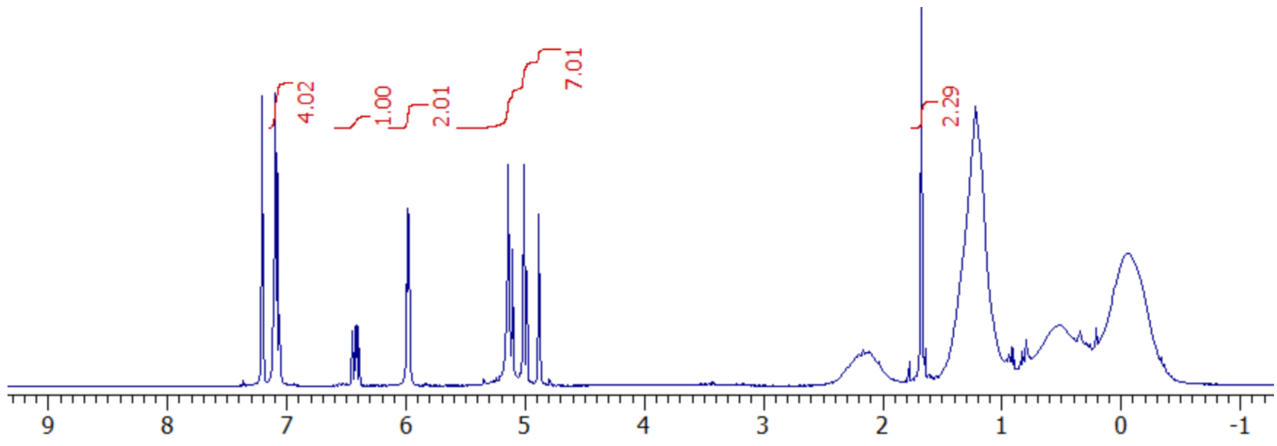

Figure S9. ^{1}H NMR spectrum after the reaction of **2a** and excess MMAO for 2 h at room temperature (500 MHz, in C_6D_6).

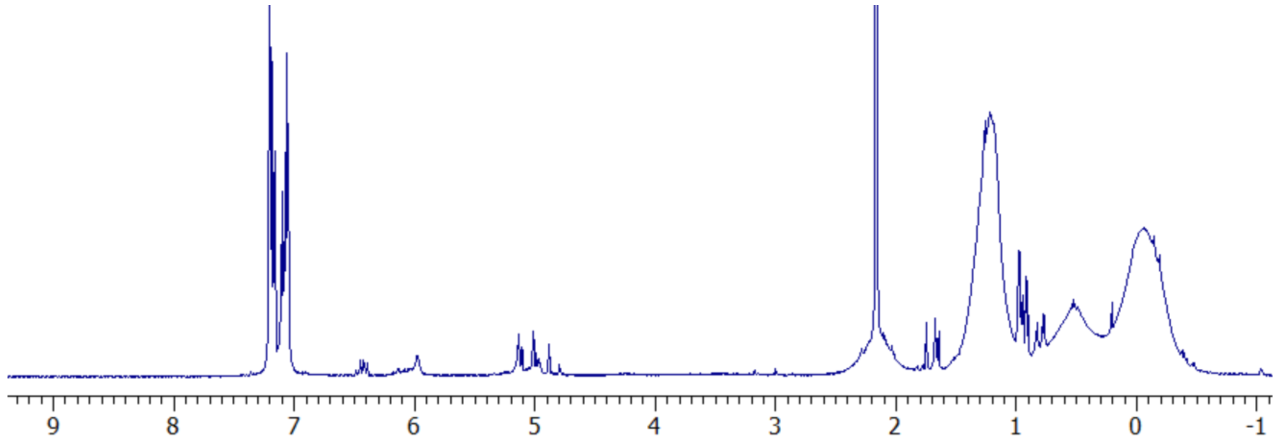

Figure S10. ^{1}H NMR spectrum after the reaction of **2a** and excess MMAO for 5 h at 45 °C (500 MHz, in C_6D_6).

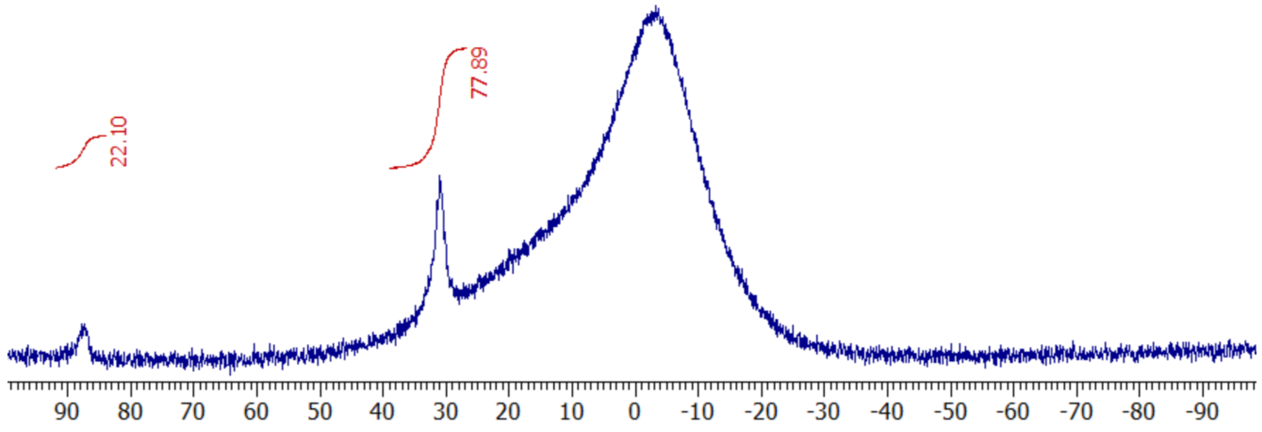

Figure S11. ^{11}B NMR spectrum after the reaction of **2a** and excess MMAO for 5 h at 45 °C (160 MHz, in C_6D_6).

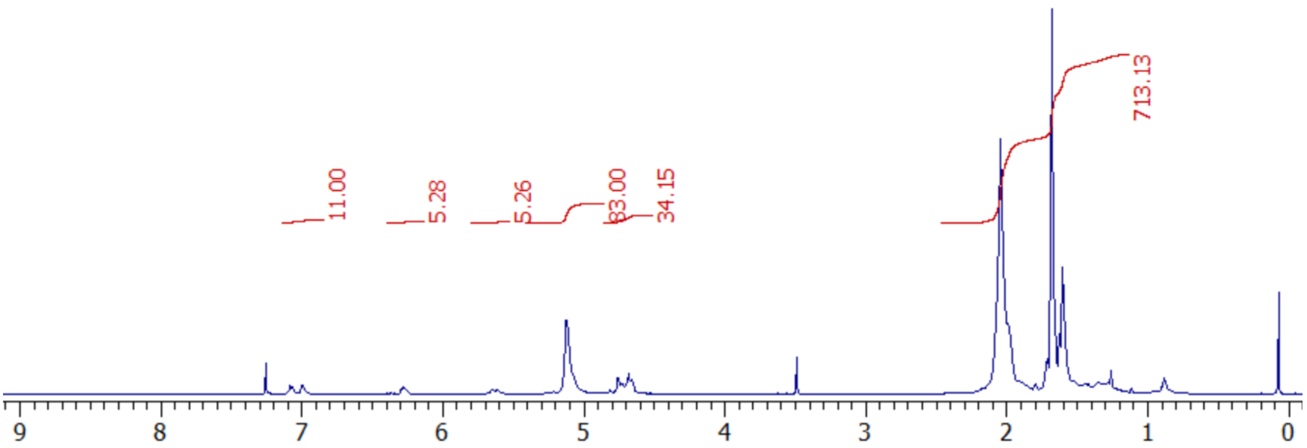

Figure S12. ^{13}C NMR spectrum of isoprene/**2a** copolymer obtained in table 1, run 1 (125 MHz, in $CDCl_3$).

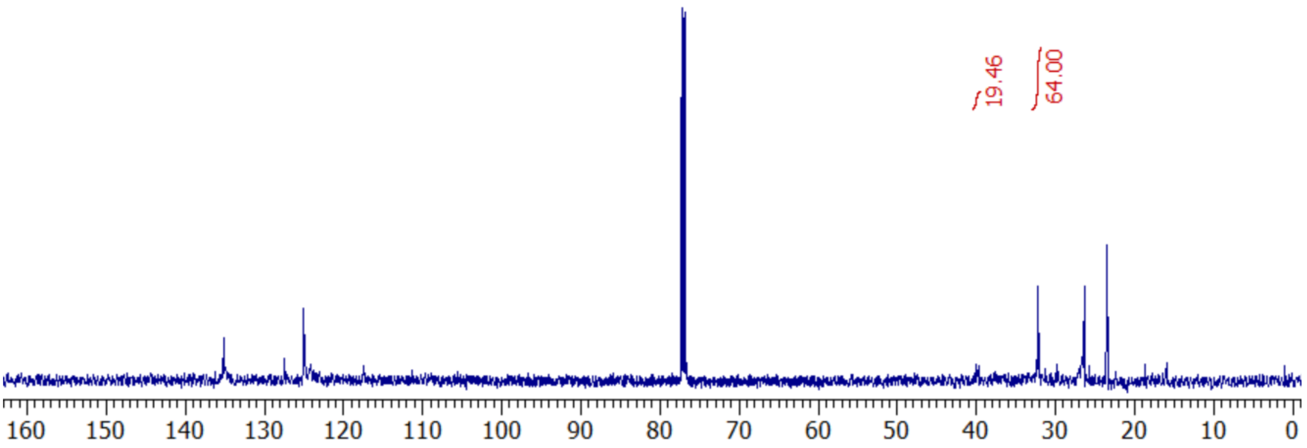

Figure S13. ^{13}C NMR spectrum of isoprene/**2a** copolymer obtained in table 1, run 1 (125 MHz, in $CDCl_3$).

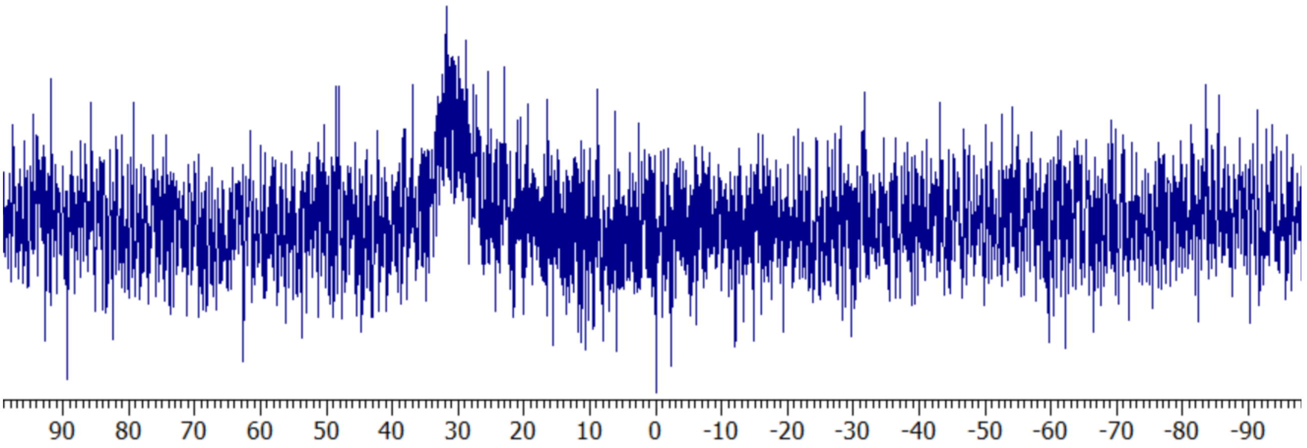

Figure S14. ^{11}B NMR spectrum of isoprene/**2a** copolymer obtained in table 2, run 2 (160 MHz, in $CDCl_3$).

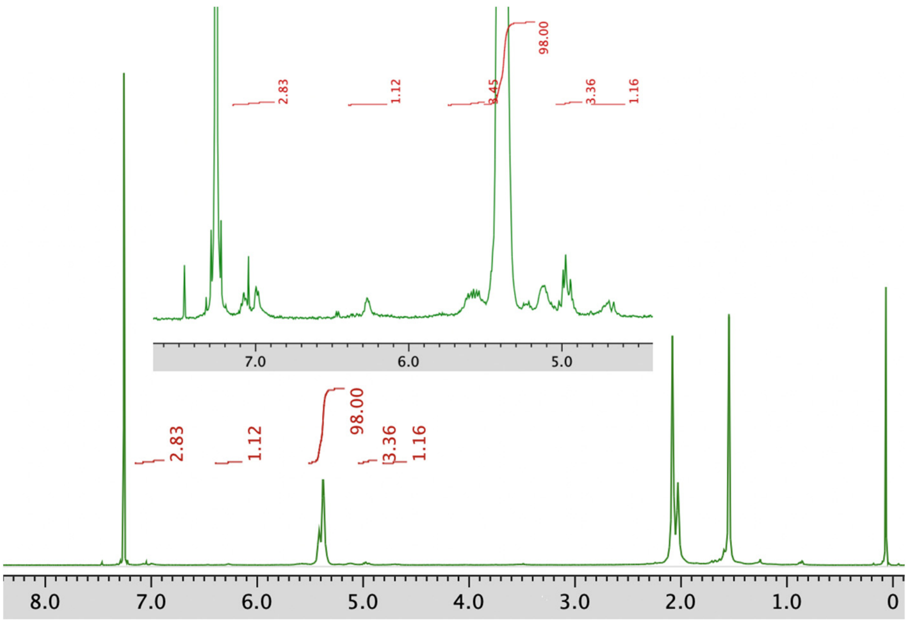

Figure S15. ^{1}H NMR spectrum of butadiene/**2a** copolymer obtained in table 1, run 5 (500 MHz, in $CDCl_3$).

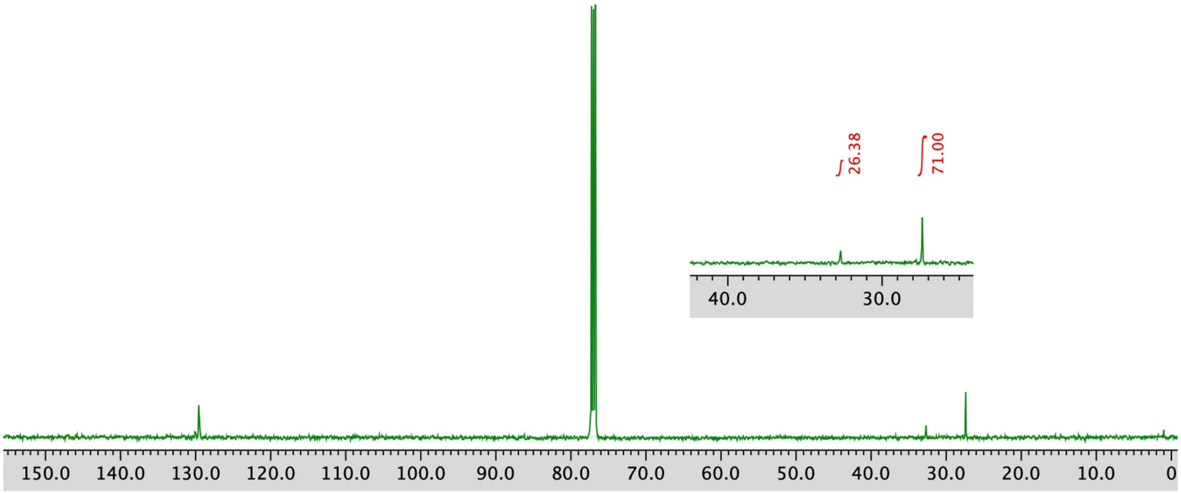

Figure S16. ^{13}C NMR spectrum of butadiene/**2a** copolymer obtained in table 1, run 5 (125 MHz, in $CDCl_3$).

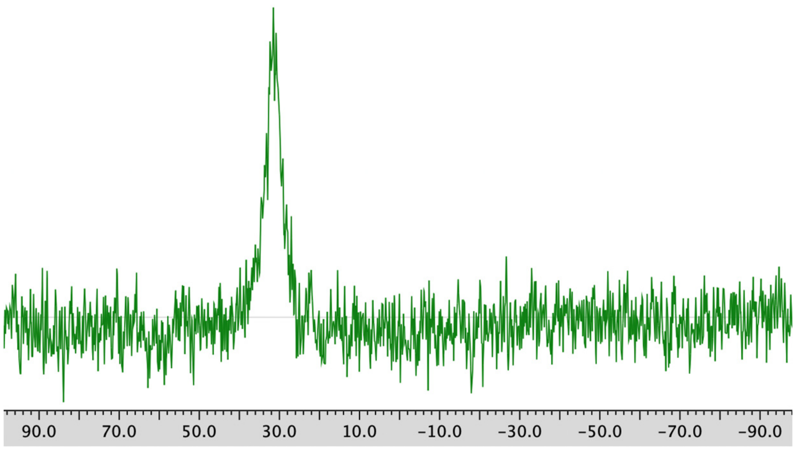

Figure S17. ^{11}B NMR spectrum of butadiene/**2a** copolymer obtained in table 2, run 2 (160 MHz, in $CDCl_3$).

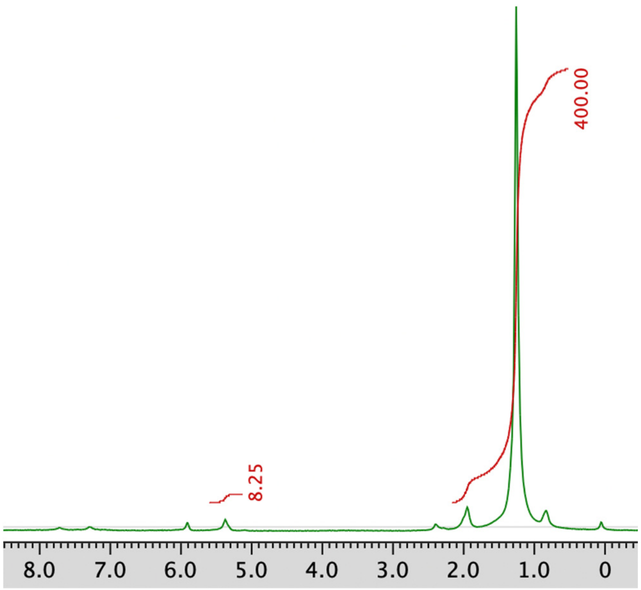

Figure S18. ^{1}H NMR spectrum of hydrogenated butadiene/**2a** copolymer (500 MHz, in $C_2D_2Cl_4$). The conversion of C=C double bond was 96 %.

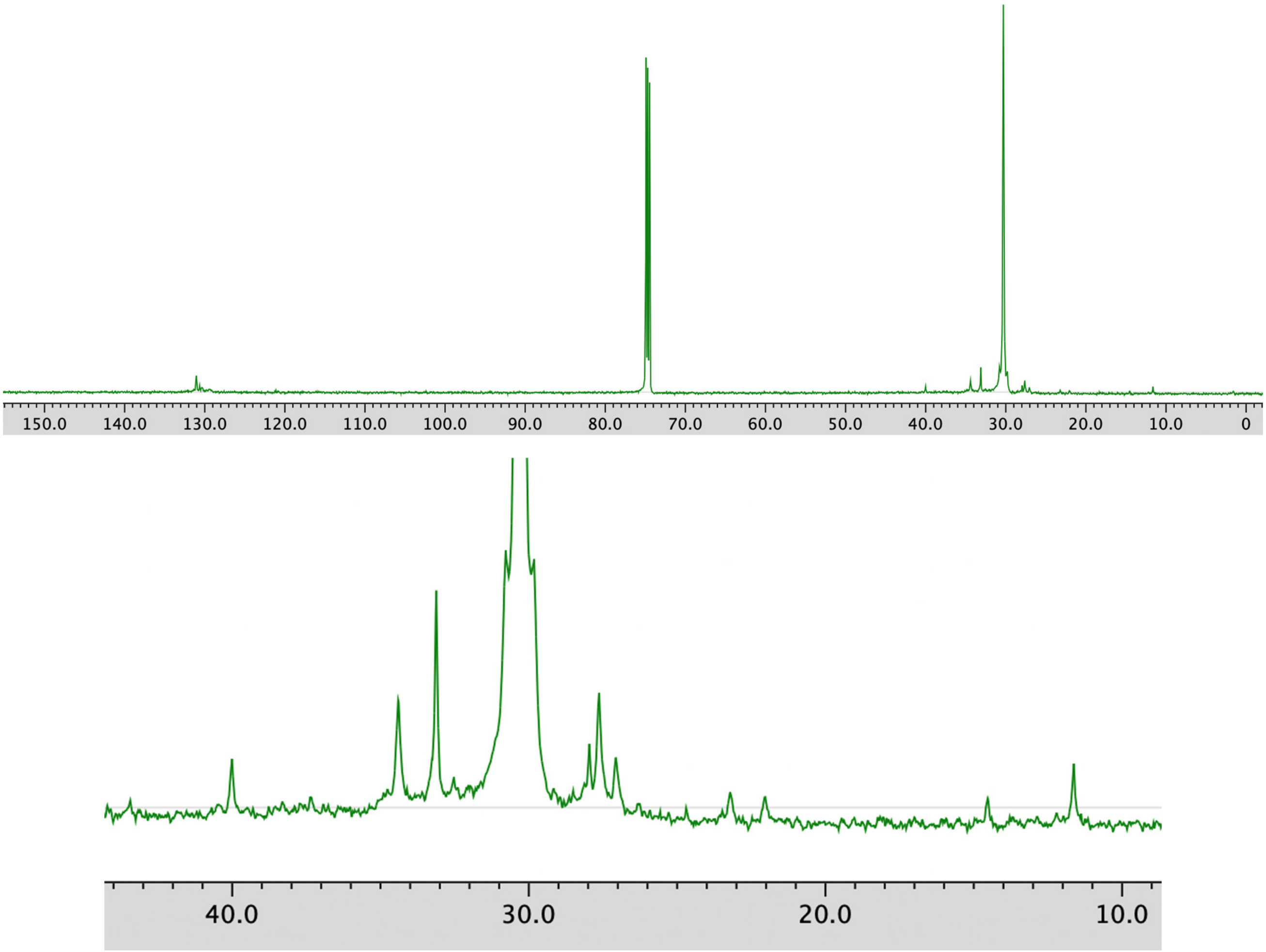

Figure S19. Full ^{13}C NMR spectrum of hydrogenated butadiene/**2a** copolymer (500 MHz, in $C_2D_2Cl_4$).
